# Gastrointestinal Stromal Tumours (GIST) of the Rectum: A Systematic Review and Meta-Analysis

**DOI:** 10.3390/curroncol30010034

**Published:** 2022-12-28

**Authors:** Shaheer I. Khan, Niall J. O’Sullivan, Hugo C. Temperley, Emanuele Rausa, Brian J. Mehigan, Paul McCormick, John O. Larkin, Dara O. Kavanagh, Michael E. Kelly

**Affiliations:** 1 Royal College of Surgeons, D02 YN77 Dublin, Ireland; 2 Department of Surgery, St James’s Hospital, D08 NHY1 Dublin, Ireland; 3 School of Medicine, Trinity College Dublin, D08 W9RT Dublin, Ireland; 4 Department of Surgery, Tallaght University Hospital, D24 NR0A Dublin, Ireland; 5 Fondazione IRCCS Istituto Nazionale dei Tumori, 20133 Milan, Italy

**Keywords:** gastrointestinal stromal tumour, rectal GIST, radical resection, local excision, chemotherapy, overall survival

## Abstract

Background: Rectal gastrointestinal stromal tumours (GISTs) have many treatment options, but uncertainty remains regarding the best treatment regimen for this rare pathology. The aim of this review is to assess the optimal management approach including timing of chemotherapy. Methods: PubMed, EMBASE, and Cochrane databases were searched for relevant articles comparing the impact of radical vs. local excision, and neoadjuvant vs. adjuvant therapy had on outcomes in the management of rectal GISTs. We specifically evaluated the influence that the aforementioned factors had on margins, recurrence, overall survival, 5-year disease-free survival, and hospital length of stay. Results: Twenty-eight studies met our predefined criteria and were included in our study, twelve of which were included in the quantitative synthesis. When comparing neoadjuvant versus adjuvant chemotherapy, our meta-analysis noted no significance in terms of margin negativity (R0) (odds ratio [OR] 2.01, 95% confidence interval [CI], 0.7–5.79, *p* = 0.20) or recurrence rates (OR 0.22, 95% CI, 0.02–1.91, *p* = 0.17). However, there was a difference in overall 5-year survival in favour of neoadjuvant therapy (OR 3.19, 95% CI, 1.37–7.40, * *p* = 0.007). Comparing local excision versus radical excision, our meta-analysis observed no significance in terms of overall 5-year survival (OR1.31, 95% CI, 0.81–2.12, *p* = 0.26), recurrence (OR 0.67, 95% CI, 0.40–1.13, *p* = 0.12), or 5-year disease-free survival (OR 1.10, 95% CI, 0.55–2.19, *p* = 0.80). There was a difference in length of hospital stay with a reduced mean length of stay in local excision group (mean difference [MD] 6.74 days less in the LE group; 95% CI, −6.92–−6.56, * *p* =< 0.00001) as well as a difference in R0 rates in favour of radical resection (OR 0.68, 95% CI, 0.47–0.99, * *p* = 0.05). Conclusion: Neoadjuvant chemotherapy is associated with improved overall 5-year survival, while local excision is associated with reduced mean length of hospital stay. Further large-volume, prospective studies are required to further define the optimal treatment regimen in this complex pathology.

## 1. Introduction

While gastrointestinal stromal tumours (GISTs) have the highest incidence among mesenchymal tumours, their presence in the rectum is rare, accounting for only 5% of all GISTs [1,2,3]. Surgery has long been the mainstay of treatment; however, the challenges faced in resection due to location and disease biology combined with advances in therapeutic modalities has led to an evolution in the management of rectal GIST [4].

Due to the challenging anatomy of the rectum, in the sense that complete tumour resection must be achieved while preserving sphincter function, a wide variety of surgical approaches have been established [5]. Historically, when tumours were large and encompassed a significant portion of the rectal lumen, there were few options available beyond radical resections such as abdominoperineal resection (APR), and in extreme cases total pelvic exenteration (TPE) [6]. While this was curative, it was associated with significant morbidity [7]. Once it was established that lymph node spread is negligible, more conservative sphincter-sparing surgeries involving local excision became more popular [8]. As surgical techniques progressed, with a shift towards minimally invasive surgery (MIS), these techniques (such as transanal minimally invasive surgery—TAMIS) were also employed to treat rectal GISTs [9].

A breakthrough in treatment options for rectal GIST was the introduction of tyrosine kinase inhibitors (TKIs) [10]. Imatinib was initially shown to reduce the risk of disease recurrence and was subsequently used as a method of reducing tumour burden preoperatively to facilitate MIS options [11]. The efficacy of neoadjuvant treatment was significant enough, in that it allowed tumours which were previously deemed unresectable to become resectable, often using sphincter-sparing methods and with lower morbidity rates [12]. Despite the development of new treatment modalities for rectal GISTs, there is an appreciable gap in the literature on the best regimen of choice due to the rarity of this condition. Our paper aims to collect and analyse data in the literature to determine the optimal treatment approach in the management of rectal GIST.

## 2. Methods

This systematic review and meta-analysis was conducted in accordance with the “Preferred Reporting Items for Systematic Reviews and Meta-Analyses” (PRISMA) extension statement for reporting of systematic reviews incorporating network meta-analyses of healthcare interventions [13]. Local institutional ethical approval was not sought as all included data were obtained from previously published studies. The study was registered with the PROSPERO database (ID: CRD42022331856) 

### 2.1. Study Selection Strategy

A formal systematic search was performed of the PubMed, EMBASE, and Cochrane databases to identify relevant titles. The following search terms were used: “gastrointestinal stromal tumours”, “GISTs”, “rect*”, “(neo)adjuvant therapy”, “local excision”, “radical excision”, and “survival”. The symbol “*” was used to allow variations on a word stem to be included in the search results. Furthermore, the following MeSH (medical subject headings) were used: GIST[MeSH], resection[MeSH], chemotherapy[MeSH], and survival[MeSH]. The grey literature (academic papers, research and committee reports, conference papers, and ongoing research) was also searched to further identify ongoing works of literature. This search was performed by two independent reviewers (S.I.K. and N.O.S.), using a predetermined search strategy that was designed by the senior authors. Details in relation to the search strategy can be found in Supplementary File S1. Manual cross-referencing of reference lists from previous review articles and included trials was undertaken. Manual removal of duplicate studies was performed before all titles were screened. Thereafter, studies considered to be appropriate had their abstracts and/or full text reviewed. Retrieved studies were reviewed to ensure inclusion criteria were met for the primary outcome at a minimum, with discordances in opinion resolved through consultation with a third author (M.K.). Data extraction was also performed by two independent reviewers (S.I.K. and N.O.S.), with study details, basic patient clinicopathological characteristics and surgical data all recorded. Furthermore, information extracted was based on the PICOTS framework (population, intervention, comparator, outcomes, timing, and setting). The final search was performed on 1 March 2022. A grey literature search was also conducted to further identify ongoing works of literature.

### 2.2. Inclusion Criteria

All original studies, irrespective of design, which compared outcomes between patient cohorts receiving any form of surgical treatment for rectal GISTs and which reported on at least one of the predefined outcomes of interest including overall survival (OS), disease-free survival (DFS), and recurrence were included in the review. In addition, we included only studies from the year 2000 onwards, studies which reported on ≥10 patients, and only studies that were written in English.

### 2.3. Exclusion Criteria

Studies published prior to the year 2000, reporting on <10 patients and in languages other than English were excluded from analysis.

### 2.4. Data Extraction and Critical Appraisal

Data extracted from each study included: year of publication, journal of publication, primary authors name, study design, period of study, number of patients included, type of surgery (primarily local resection vs. radical resection), use of neoadjuvant and adjuvant therapy, and long-term outcomes (overall survival, disease-free survival, and recurrence). Additional data collected included margin status, length of stay in hospital, and intraoperative tumour rupture rates. 

Data were collected by two reviewers independently, using the following headings: study details, study design, population, intervention, comparison groups, and outcomes. Conflicts between the two reviewers were resolved following a discussion and final decision by the senior author.

The quality of the studies included in this systematic review was assessed using the Newcastle Ottawa scale. Furthermore, the certainty of evidence was assessed using the grading of recommendations, assessment, development, and evaluations (GRADE) tool for grading quality of evidence [14]. The quality score rating was determined for each publication and recorded. 

### 2.5. Outcomes of Interest

The following outcomes were used in the analysis to compare the effect of neoadjuvant versus adjuvant therapy and local excision versus radical excision.

#### 2.5.1. Primary Outcomes

The primary outcome of interest was the impact that surgical strategy had on survival outcomes (5-year overall survival, 5-year disease-free survival, recurrence and negative margins (R0) rates for the management of rectal GISTs). Specifically comparing neoadjuvant versus adjuvant therapy and local excision versus radical excision.

#### 2.5.2. Secondary Outcomes

Alongside the primary outcomes, length of stay and intraoperative tumour rupture between local excision versus radical excision was analysed.

### 2.6. Statistical Analysis

Statistical analysis was performed using Revman Statistical Software (Ver. 5, Copenhagen, Denmark). Binary outcome data were reported as odd ratios (OR) and 95% confidence interval (95% CI) were estimated using the Mantel–Haenszel method. For continuous data, mean differences and 95% CI were estimated using inverse variance weighting. Outcome measures (mean + standard deviation and median + interquartile range) were recorded. If needed, outcome variables (mean and SD) were estimated from the median and range using formula described by Hozo et al. [15]. Heterogeneity was assessed by I-squared statistics, with >50% being considered as considerable heterogeneity. Statistical significance was attributed to *p*-value < 0.05. 

## 3. Results:

### 3.1. Search Results

Our initial search produced 1797 results in total. After removing duplicate articles, the remaining 552 articles were screened. From these articles, 51 articles had their abstracts reviewed for eligibility, of which sixteen articles were removed due to not meeting the inclusion/exclusion criteria. Ultimately, we identified 28 studies which met our predefined criteria and reported our desired outcomes, and 12 of these studies were included in our meta-analysis (Supplementary File S1).

### 3.2. Methodological Characteristics and Quality of Studies

All 28 of the identified studies are retrospective, cohort studies with more than ten patients included [1,5,16,17,18,19,20,21,22,23,24,25,26,27,28,29,30,31,32,33,34,35,36,37,38,39,40,41]. Only articles published in English were accepted. A summary of Table 1 summarises the methodological characteristics of the included studies. The methodological quality of the included studies was generally good and can be found in Supplementary Table S1. Nine studies achieved a rating of 7 or higher on the Newcastle Ottawa Scale (NOS), meeting criteria for ”high quality” studies. The GRADE certainty of evidence ranged from very low to low and is presented in the Supplementary Table S2.

### 3.3. Participant Characteristics

The total number of participants who underwent surgery from the twenty-eight studies included was 1654. Of these patients, 813 underwent local excision, while 740 patients underwent radical excision procedures, and it was unspecified in 101 patients. Overall, 17/28 reported on initial diagnosis tumour size, with an overall mean of 5.32 cm (±3.77). Overall, 8/28 studies (293 patients) report on which specific surgical approach was undertaken. Laparotomy was most commonly performed (59.0% (173/293) of patients), followed by transanal (29.4% (86/293)) and laparoscopic (11.6% (34/293)). A summary of surgical details, including tumour size and margin status data, can be found in Table 2. 

### 3.4. Neoadjuvant Versus Adjuvant Outcomes

#### 3.4.1. Chemotherapy Characteristics

Overall, 23 studies reported on neoadjuvant chemotherapy. In total, 40.9% (539/1316) patients underwent neoadjuvant chemotherapy. All studies which reported on neoadjuvant therapy utilised Imatinib (99.6% (537/539)), with only 1/23 studies reporting Sunitinib (1/539) and Adriamycin plus ifosfamide (1/539) use. The overall median time of neoadjuvant therapy was 7.7 months (range: 1–102 months). Overall, 22 studies reported on adjuvant chemotherapy, with 39.4% (491/1246) of patients undergoing adjuvant therapy. All studies that reported on adjuvant therapy utilized Imatinib only. The overall median time of adjuvant therapy was 18 months (range: 0–112 months). A summary of chemotherapy details can be found in Table 3.

#### 3.4.2. Recurrence

**Figure 1:**
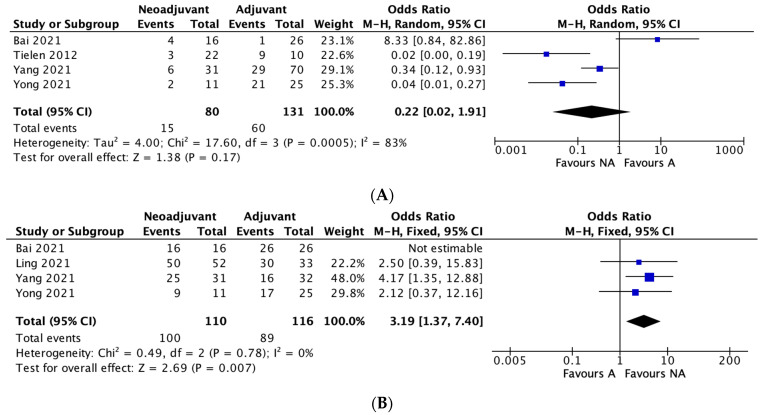

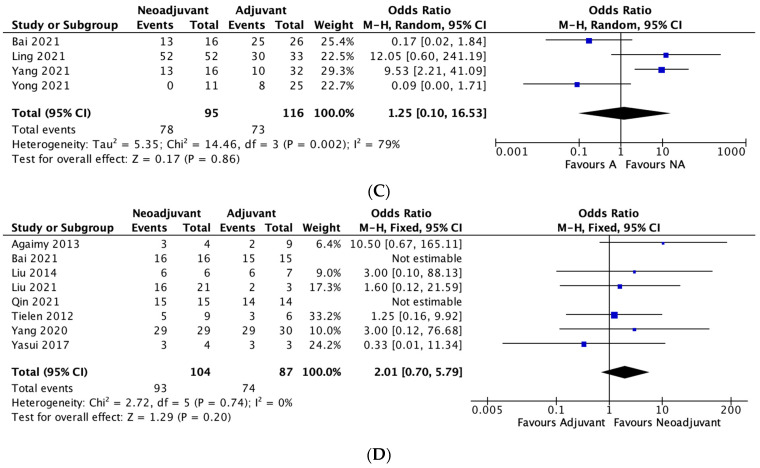
(**A**–**D**): Neoadjuvant vs. adjuvant chemotherapy meta-analysis outcomes. (**A**) Recurrence, (**B**) Five-year overall survival, (**C**) Five-year disease-free survival, (**D**) Negative margin (R0) rates.

Four studies reported recurrence rates between neoadjuvant and adjuvant therapy groups. The recurrence rate was 18.75% in the neoadjuvant group and 45.8% in the adjuvant therapy group. A meta-analysis of the included studies using an M-H random effects model showed no significant difference between the two groups in regard to recurrence rates (OR 0.22, 95% CI, 0.02–1.91, *p* = 0.17), with significant heterogeneity between studies (I^2^ = 83%) (Figure 1A).

#### 3.4.3. Five-Year Overall Survival

Four studies reported overall 5-year survival rates between the two groups. The 5-year survival rate was 90.9% in the neoadjuvant group and 76.7% in the adjuvant group. A meta-analysis of the included studies using an M-H fixed effects model showed a significant difference between the two groups in terms of overall 5-year survival rates, in favour of neoadjuvant therapy (OR 3.19, 95% CI, 1.37–7.40, * *p* = 0.007), with no heterogeneity between studies (I^2^ = 0%). (Figure 1B).

#### 3.4.4. Five-Year Disease-Free Survival

Four studies reported on disease-free survival rates between the two groups. The 5-year disease-free survival rate was 82.1% in the neoadjuvant group and 62.9% in the adjuvant group. The meta-analysis demonstrated no significant difference between the two groups in terms of 5-year disease-free survival rates (OR 1.25, 95% CI, 0.10–16.53, *p* = 0.86), with significant heterogeneity between studies (I^2^ = 79%). (Figure 1C)

#### 3.4.5. Negative Margin (R0) Rates

Eight studies reported on R0 rates between the two groups. The R0 rate was 89.4% in the neoadjuvant group and 85.1% in the adjuvant group. The meta-analysis demonstrated no significant difference between the two groups in terms of R0 rates (OR 2.01, 95% CI, 0.7–5.79, *p* = 0.20), with no heterogeneity between studies (I^2^ = 0%). (Figure 1D)

### 3.5. Local Excision vs. Radical Resection Outcomes

#### 3.5.1. Recurrence

**Figure 2:**
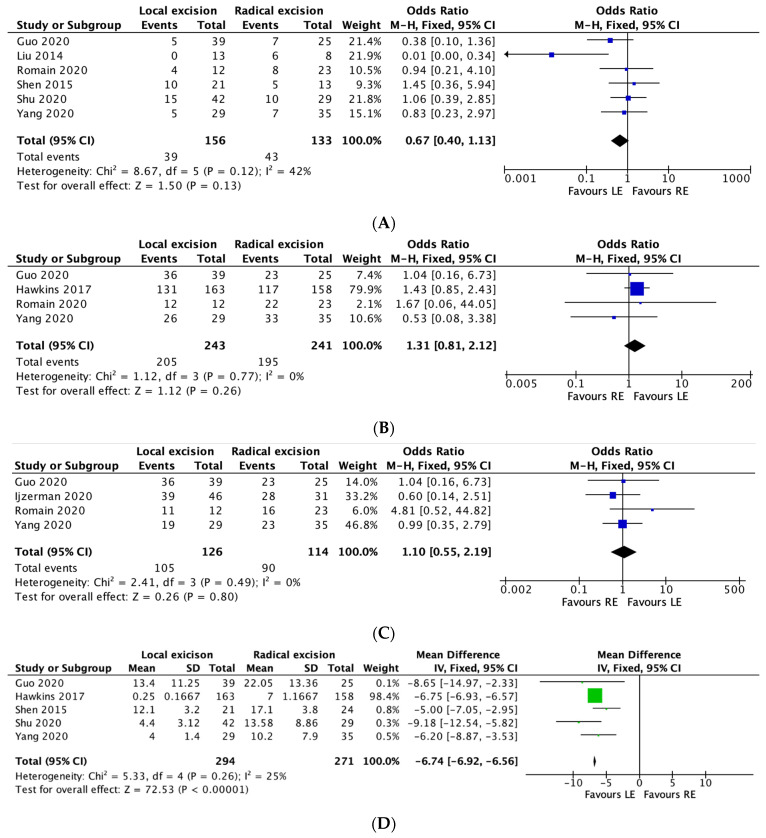

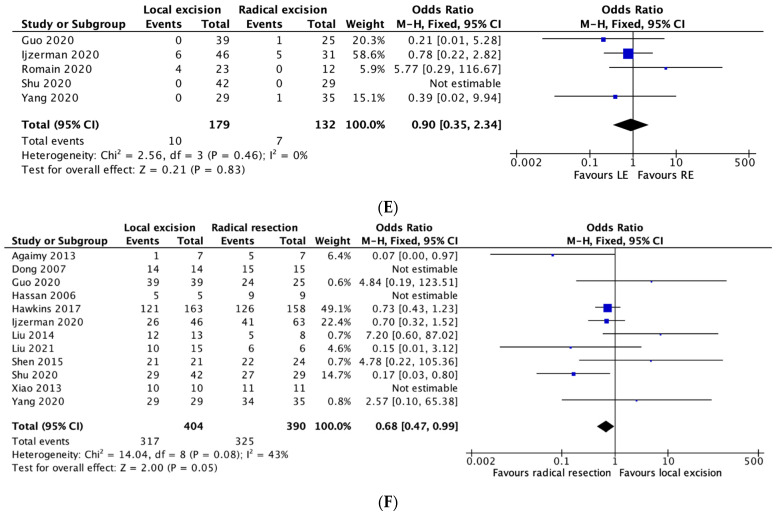
(**A**–**F**): Local excision vs. radical excision meta-analysis results. (**A**) Recurrence, (**B**) Five-year Overall Survival, (**C**) Five-year Disease-free Survival, (**D**) Length of Stay, (**E**) Intraoperative Tumour Perforation, (**F**) R0 Rates.

Six studies reported on overall recurrence rates between the two groups. The overall recurrence rate was 25% in the local excision group and 32.3% in the radical excision group. A meta-analysis performed using the M-H fixed effects model demonstrated no significant difference between the two groups in terms of overall recurrence (OR 0.67, 95% CI, 0.40–1.13, *p* = 0.12), with moderate heterogeneity between studies (I^2^ = 42%). (Figure 2A).

#### 3.5.2. Five-Year Overall Survival:

Four studies reported overall 5-year survival rates. The rate was 84.4% in the local excision group and 80.9% in the radical excision group. The meta-analysis noted no significant difference between the two groups in terms of 5-year survival rates (OR 1.31, 95% CI, 0.81–2.12, *p* = 0.26), with no heterogeneity between studies (I^2^ = 0%). (Figure 2B)

#### 3.5.3. Five-Year Disease-Free Survival:

Four studies reported 5-year disease-free survival between the two groups. The 5-year disease-free survival rate was 83.3% in the local excision group and 78.9% in the radical excision group. The meta-analysis demonstrated no significant difference between the two groups in relation to 5-year disease-free survival rates (OR 1.10, 95% CI, 0.55–2.19, *p* = 0.80), with no heterogeneity between studies (I^2^ = 0%). (Figure 2C).

#### 3.5.4. Length of Stay

Five studies reported on length of stay (days) between the two groups. The meta-analysis performed using the fixed-effects model demonstrated a reduced length of stay in the local excision group (MD 6.74 days less in the LE group; 95% CI, −6.92–−6.56, * *p* =< 0.00001), with low heterogeneity between studies (I^2^ = 25%). (Figure 2D).

#### 3.5.5. Intraoperative Tumour Perforation:

Five studies reported on tumour perforation rates between the two groups. The tumour perforation rate was 5.6% in the local excision group and 5.3% in the radical excision group. Meta-analysis revealed no significant difference between the two groups in terms of tumour perforation rates (OR 0.90, 95% CI, 0.35–2.34, *p* = 0.83), with no heterogeneity between studies (I^2^ = 0%). (Figure 2E)

#### 3.5.6. R0 Rates

Twelve studies reported R0 rates between the two groups. The R0 rate was 78.5% in the local excision group and 83.3% in the radical resection group. Meta-analysis demonstrated a significant difference in R0 rates between the two groups, in favour of radical resection (OR 0.68, 95% CI, 0.47–0.99, * *p* = 0.05), with moderate heterogeneity reported across the studies (I^2^ = 43%). (Figure 2F)

## 4. Discussion

Our systematic review observed how the timing of chemotherapy and radicalness of surgery impacts on the management of rectal GISTs. While case reports on the topic are common, large prospective studies are limited, and there is a considerable lack of high-level evidence regarding the optimal management of this rare entity. To the best of our knowledge, our study is the first meta-analysis comparing local and radical excision, as well as neoadjuvant vs. adjuvant therapy in the management of rectal GISTs. 

Our review demonstrated no significant difference between neoadjuvant and adjuvant therapy in terms of recurrence and 5-year disease-free survival rates. It did however reveal a significant overall survival benefit in favour of neoadjuvant therapy. These results demonstrate that the use of neoadjuvant chemotherapy in rectal GISTs patients might not play a role in preventing local recurrence, but potentially does impact overall survival (90.9% in the neoadjuvant group vs. 76.7% in the adjuvant group). Similarly, when comparing local vs. radical excision there was no difference in 5-year disease free survival or recurrence rates. In addition, the method of excision did not influence overall survival either (84.4% in the local excision group vs. 80.9% in the radical excision group). However, local excision did have some minor benefits such as reduced hospital stay. These results suggest that local excision when applicable should be utilised as it does not have inferior oncological outcomes, and would likely reduce overall stoma rate or morbidity that is associated with radical rectal surgery. 

Recent studies have highlighted the benefits of neoadjuvant therapy in tumour down-sizing resulting in R0 rates after resection as well as improved anal sphincter preservation [12]. This can often be challenging, as rectal GIST’s can be of a large size within the confines of a narrow pelvis [5]. Unfortunately, data in this study were limited in terms of tumour response to neoadjuvant therapies; however, they did show an overall survival benefit with neoadjuvant treatment. The benefit in overall survival may be attributed to the fact that neoadjuvant therapy in general is associated with improved patient compliance when compared to that of adjuvant therapy [42]. Neoadjuvant radiotherapy has also been reported to have fewer significant side effects [42]. However, due to significant heterogeneity between studies in terms of neoadjuvant and adjuvant regimens, it would be prudent to suggest the benefit of one modality over another without further large-scale randomized trials. Due to variation in the chemotherapeutic regimens across different studies, there is a lack of comparability and results should therefore be interpreted with caution.

Regardless of treatment choice, it is imperative to take patient quality of life (QoL) into consideration when selecting patients for radical therapy. Our data provide important information regarding the non-inferiority of local excision in terms of recurrence or survival, which may reduce the necessity of radical resection and its associated morbidity moving forward. To date, there is a considerable lack of QoL data from patients being managed for rectal GISTs. Additionally, the introduction of TKIs, such as Imatinib, has revolutionized the management of primary and recurrent diseases, particularly via tumour downsizing and a reduction in mitotic activity, morbidity, and recurrence [12]. This method of chemo-reduction is particularly useful for distal tumours, where conventional resection may compromise the anal sphincter [16], resulting in significant long-term morbidity. Few studies have directly compared quality of life in patients undergoing radical or sphincter-preserving surgery; however, evidence does point towards improved functional outcomes without compromising oncological outcomes [43,44].

The authors acknowledge that the review does have some limitations. The rarity of this condition, combined with the heterogeneity in management between studies, prevents large-volume analysis. It is unlikely that an RCT could ever recruit adequately and compare several treatment approaches. Despite this, our study provides important data for the shared decision-making process. Future studies should also focus on quality-of-life outcomes in patients undergoing local or radical excision of rectal GIST, incorporating outcome data on surgical approach (open versus minimally invasive platforms). Nonetheless, our study will impact clinical practice by allowing clinicians and surgeons to counsel patients on the optimal managements options and inform them on expected outcomes.

## Figures and Tables

**Table 1:** Methodological characteristics of the included studies.

Study Name	Journal	Year Published	Study Period	Type of Study	N of Rectal GISTs Patients
Yong et al. [16]	*International Journal of Clinical Oncology*	2021	1996 to 2017	Retrospective Cohort Study	29
Yang et al. [17]	*BMC Surgery*	2021	2002 to 2020	Retrospective Cohort Study	101
Qin et al. [18]	*Annals of Translational Medicine*	2021	2008 to 2018	Retrospective study	17
Liu et al. [19]	*Cancer Management and Research*	2021	2010 to 2019	Retrospective Cohort Study	21
Ling et al. [20]	*Journal of Surgical Oncology*	2021	2007 to 2018	Retrospective case–control study	68
Emoto et al. [21]	*Journal of Gastrointestinal Surgery*	2021	2008 to 2017	Retrospective Cohort Study	20
Bai et al. [22]	*Surgical Endoscopy*	2021	2006 to 2017	Retrospective Cohort Study	42
Yang et al. [23]	*Annals of Translational Medicine*	2020	2008 to 2018	Retrospective Cohort Study	64
Shu et al. [24]	*International Journal of Surgery*	2020	2004 to 2017	Retrospective Cohort Study	71
Romain et al. [25]	*Journal of Surgical Oncology*	2020	2001 to 2013	Retrospective Cohort Study	35
Ijzerman et al. [26]	*European Journal of Surgical Oncology*	2020	2009 to 2018	Retrospective, multicentre, international cohort study	155 total surgery/ 109 with data analysis
Guo et al. [27]	*International Journal of Surgery*	2020	2008 to 2019	Retrospective Cohort Study	64
Stuart et al. [28]	*Journal of Surgical Oncology*	2019	1976 to 2017	Retrospective review	48
Zhu et al. [29]	*Journal of Gastrointestinal Oncology*	2018	2006 to 2013	Retrospective Cohort Study	282
Yasui et al. [30]	*Surgery Today*	2017	2003 to 2007	Retrospective Cohort Study	24
Hawkins et al. [31]	*Annals of Surgical Oncology*	2017	1998 to 2012	Retrospective Cohort Study	321
Cavnar et al. [32]	*Annals of Surgical Oncology*	2017	1982 to 2016	Retrospective review	47
Zanwar et al. [33]	*Indian Journal of Gastroenterology*	2016	2005 to 2015	Cohort Study	18
Wilkinson et al. [34]	*British Journal of Surgery*	2015	2001 to 2013	Retrospective Cohort Study	13
Shen et al. [35]	*Neoplasma*	2015	2005 to 2014	Retrospective Cohort Study	45
Liu et al. [1]	*Journal of Surgical Oncology*	2014	2002 to 2010	Retrospective review	21
Huynh et al. [36]	*BMC Cancer*	2014	1991 to 2011	Retrospective Cohort Study	41
Xiao et al. [37]	*Journal of Gastrointestinal Surgery*	2013	1986 to 2010	Retrospective Cohort Study	21
Tielen et al. [5]	*Journal of Surgical Oncology*	2013	1990 to 2011	Retrospective Cohort Study	32
Agaimy et al. [38]	*International Journal of Colorectal Disease*	2013	2000 and 2011	Retrospective multicentre study	15
Dong et al. [39]	*Scandinavian Journal of Gastroenterology*	2007	1997 to 2005	Retrospective Cohort Study	29
Hassan et al. [40]	*Diseases of the Colon and Rectum*	2006	1979 to 2004	Retrospective Cohort Study	14
Changchien et al. [41]	*Diseases of the Colon and Rectum*	2004	1979 to 1999	Retrospective Cohort Study	42

**Table 2:** Summary of Surgical Detail.

Study Name	Total N Patients	Tumour Size at Diagnosis (cm)-Mean	Local Excision	Radical Excision	Surgical Approach	Margin Status		
						R0/%	R1/%	R2/%
Yong et al. [16]	29	-	-	29	Transanal 7/29 Laparoscopic 2/29 Open 20/29	15 of 29/51.7%	10 of 29/34.5%	-
Yang et al. [17]	101	6.18 ± 3.02	95	6	Transanal 5/101 Laparoscopic 14/101 Open 82/101	97 of 101/96%	4 of 101/4%	
Qin et al. [18]	17	6.4 ± 2.2	-	17	-	17 of 17/100%	0 of 17/0%	
Liu et al. [19]	21	4.96 ± 3.02	15	6	-	16 of 21/76.2%	5 of 21/23.8%	-
Ling et al. [20]	68	-	50	14	-	-		
Emoto et al. [21]	20	6.5 ± 3	4	16	Transanal 4/20 Laparoscopic 15/20 Open 1/20	17 of 20/85%	3 of 20/15%	
Bai et al. [22]	42	2.87 ± 1.61	42	-	Transanal 42/42 Laparoscopic 0/42 Open 0/42	42 of 42/100%	0 of 42/0%	
Yang et al. [23]	64	-	29	35	Transanal 29/64 Laparoscopic N/a Open N/a Nontransanal 35/64	63 of 64/98.4%	1 of 64/1.6%	
Shu et al. [24]	71	-	42	29	-	56 of 71/78.9%	15 of 71/21.2%	0 of 71/0%
Romain et al. [25]	35	-	35		Transanal 9/35 Laparoscopic 3/35 Open 23/35	30 of 35/85.7%	4 of 35/11.4%	1 of 35/2.9%
Ijzerman et al. [26]	109	6.5 ± 3.67	46	63	-	67 of 109/61.5%	31 of 109/28.4%	10 of 109/9.2%
Guo et al. [27]	64	-	39	25	-	63 of 64/98.4%	1 of 64/1.6%	
Stuart et al. [28]	48	-	48		-	-		
Zhu et al. [29]	282	-	144	138	-	219 of 282/77.7%		3 of 282/1.1%
Yasui et al. [30]	24	4.8 ± 2.38	9	14	Transanal 1/24 Laparoscopic 0/24 Open 23/24	22 of 24/91.7%	1 of 24/4.2%	1 of 24/4.2%
Hawkins et al. [31]	321	4.0 ± 0.8	163	158	-	247 of 321/76.9%	74 of 321/23.1%	
Cavnar et al. [32]	47	-	23	24	-	33 of 47/70.2%	12 of 47/25.5%	2 of 47/4.3%
Zanwar et al. [33]	18	6 ± 2.45	4	14	-	17 of 18/94.4%	0 of 18/0%	1 of 18/5.6%
Wilkinson et al. [34]	13	7⋅6 ± 2.6	13		-	12 of 13/92.3%	0 of 13/0%	1 of 13/7.7%
Shen et al. [35]	45	6.0 ± 3	21	24	-	43 of 45/95.6%	2 of 45/4.4%	0 of 45/0%
Liu et al. [1]	21	6.53 ± 2.45	13	8	-	17 of 21/81%	4 of 21/19%	
Huynh et al. [36]	41	6.3 ± 3.1	18	23	-	22 of 41/53.7%	13 of 41/31.7%	4 of 41/9.8%
Xiao et al. [37]	21	7.5 ± 6.4	10	11	-	21 of 21/100%	0 of 21/0%	
Tielen et al. [5]	32	9.2 ± 12.75	7	25	Transanal 2/32 Laparoscopic N/a Open N/a Nontransanal 30/32	24 of 32/75%	6 of 32/18.8%	2 of 32/6.3%
Agaimy et al. [38]	15	4.8 ± 2.17	7	8	-	6 of 15/40%	2 of 15/13.3%	6 of 15/40%
Dong et al. [39]	29	5.0 ± 4.4	14	15	Transanal 14/29 Laparoscopic 0/29 Open 15/29	29 of 29/100%	0 of 29/0%	
Hassan et al. [40]	14	-	5	9	Transanal 4/14 Laparoscopic 0/14 Open 10/14	14 of 14/100%	0 of 14/0%	
Changchien et al. [41]	42	-	13	29	-	-		

**Table 3:** Summary of Chemotherapy Details.

Study Name	Neo-Adjuvant Therapy			Adjuvant Therapy		
	No. Patients	Type	Duration (Median (Month)) (Range)	No. Patients	Type	Duration (Median (Month)) (Range)
Yong et al. [16]	11/36	Imatinib	8.8 (4.5–33.9)	-	-	-
Yang et al. [17]	31/101	Imatinib	-	49/101	Imatinib	-
Qin et al. [18]	15/17	Imatinib	-	14/17	Imatinib	1 (1–8)
Liu et al. [19]	21/36	Imatinib	17	10/21	Imatinib	17
Ling et al. [20]	52/85	Imatinib	6.9 (1.0–58.9)	40/68	Imatinib	-
Emoto et al. [21]	16/20	Imatinib	7 (4–11)	11/20	Imatinib	35 (11–108)
Bai et al. [22]	16/42	Imatinib	6	15/42	Imatinib	18 (7–36)
Yang et al. [23]	29/64	Imatinib	-	30/64	Imatinib	-
Shu et al. [24]	23/71	Imatinib	7.0 (6–12)	21/71	Imatinib	-
Romain et al. [25]	22/35	Imatinib	9 (4–14)	21/35	Imatinib	-
Ijzerman et al. [26]	78/109	Imatinib	10 (1–102),	70/109	Imatinib	25 (0–112).
Guo et al. [27]	29/64	Imatinib	-	30/64	Imatinib	-
Stuart et al. [28]	8/48	Imatinib	-	22/48	Imatinib	-
Zhu et al. [29]	-	-	-	-	-	-
Yasui et al. [30]	4/24		2.5 (1–6)	3/24	Imatinib	-
Hawkins et al. [31]	86/321	Imatinib	-	82/321	Imatinib	-
Cavnar et al. [32]	21/47	Imatinib	7.7 (3–62) 22	12/47	Imatinib	2.8 (0.1–6.5)
Zanwar et al. [33]	16/23	Imatinib	15 (3–84)	-	-	-
Wilkinson et al. [34]	15/19	Imatinib	18 (11–44)	7/19	Imatinib	-
Shen et al. [35]	3/45	Imatinib	-	13/45	Imatinib	18 (3–46).
Liu et al. [1]	5/21	Imatinib	6 (6–8)	8/21	Imatinib	-
Huynh et al. [36]	12/41	Imatinib	7 (2–12).	11/41	Imatinib	7 (2–41)
Xiao et al. [37]	-	Imatinib	-	4/21	Imatinib	-
Tielen et al. [5]	22/32	Imatinib	9 (2–53)	9/32	Imatinib	-
Agaimy et al. [38]	4/15	Imatinib (2/4)Sunitinib (1/4)Adriamycin + Holoxan (1/4)	-	9/15	Imatinib	-
Dong et al. [39]	-	-	-	-	-	-
Hassan et al. [40]	-	-	-	-	-	-
Changchien et al. [41]		-	-	-	-	-

## Supplementary Materials

The following supporting information can be downloaded at: https://www.mdpi.com/article/10.3390/curroncol30010034/s1, File S1: Study selection. A PRISMA Flowchart of the selection of relevant publications included in this review; Table S1: Newcastle Ottawa Scale (NOS) risk of bias assessment for non-randomised studies; Table S2: GRADE Certainty of Evidence table.
