# Supplementary material for: Gastrointestinal Stromal Tumours (GIST) of the Rectum: A Systematic Review and Meta-Analysis
Source: Curr Oncol. 2022 Dec 28;30(1):416–29. doi: 10.3390/curroncol30010034 (PMC9857930; doi:10.3390/curroncol30010034)
Supplement: Supplementary file 1 [file curroncol-30-00034-s001.zip › curroncol-2076596-supplementary.pdf]

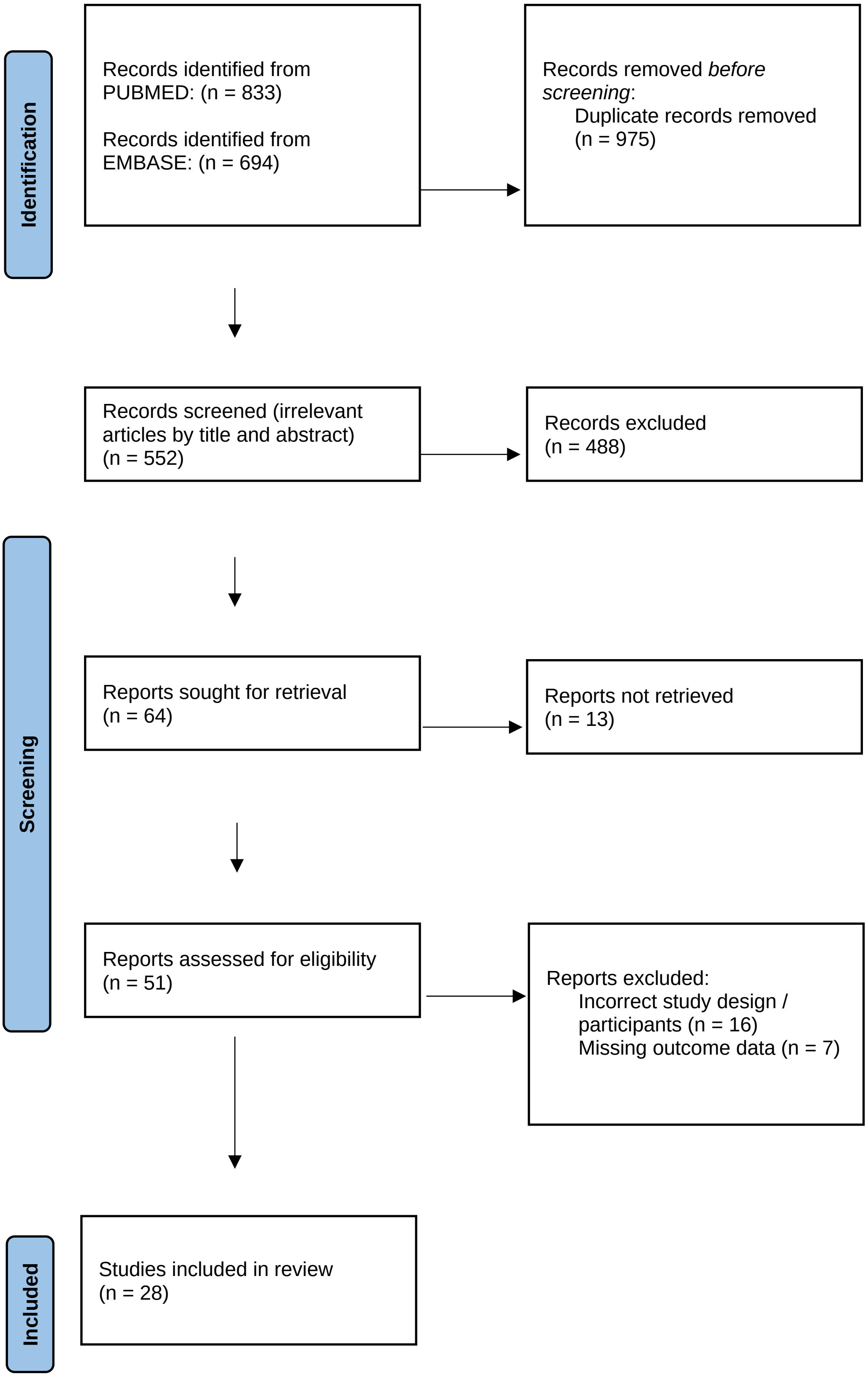

Supplementary File S1: Study selection. A PRISMA Flowchart of the selection of relevant publications included in this review

Study Name	Year	Selection Representativeness of the Exposed Cohort	Sample size (<25=no star)	Non-respondents	Ascertainment of the exposure	Comparability (The subjects in different outcome groups are comparable)	Outcome (assessment of outcome)	Statistical test	Follow Up Period (>5 Years)	Quality
Yong et al.[16]	2021	✷	✷	✷	✷	✷✷	✷	✷	✷	9
Yang et al.[17]	2021	✷	✷	✷	✷	✷✷	✷	✷	✷	9
Qin et al.[18]	2021	✷	/	/	✷	✷	✷	/	✷	5
Liu et al.[19]	2021	✷	/	✷	✷	/	✷	/	✷	5
Ling et al.[20]	2021	✷	✷	✷	✷	✷	/	✷	✷	7
Emoto et al.[21]	2021	✷	/	✷	/	✷	/	/	✷	4
Bai et al.[22]	2021	✷	✷	✷	✷	✷	✷	✷	✷	8
Yang et al.[23]	2020	✷	✷	✷	/	✷	✷	✷	✷	7
Shu et al.[24]	2020	✷	✷	✷	✷	✷✷	✷	✷	✷	9
Romain et al.[25]	2020	✷	✷	/	/	✷✷	/	✷	✷	6
Ijzerman et al.[26]	2020	✷	✷	✷	/	✷✷	✷	✷	/	7
Guo et al.[27]	2020	✷	✷	✷	✷	✷	/	✷	✷	7
Stuart et al.[28]	2019	✷	✷	✷	/	/	✷	/	✷	5
Zhu et al.[29]	2018	✷	✷	✷	/	/	/	/	✷	4
Yasui et al.[30]	2017	✷	/	✷	/	/	✷	/	✷	4
Hawkins et al.[31]	2017	✷	✷	✷	/	✷	/	✷	✷	6
Cavnar et al.[32]	2017	✷	✷	✷	✷	/	/	/	✷	5
Zanwar et al.[33]	2016	✷	/	/	✷	/	✷	/	/	3
Wilkinson et al.[34]	2015	✷	/	✷	✷	/	✷	/	/	4
Shen et al.[35]	2015	✷	✷	✷	✷	✷✷	/	✷	✷	8
Liu et al.[1]	2014	✷	/	✷	✷	✷	/	✷	✷	6
Huynh et al.[36]	2014	✷	✷	✷	/	/	✷	/	/	4
Xiao et al.[37]	2013	✷	/	✷	✷	/	✷	/	✷	5
Tielen et al.[5]	2013	✷	✷	/	/	✷	✷	✷	/	5
Agaimy et al.[38]	2013	✷	/	✷	✷	/	✷	/	/	4
Dong et al.[39]	2007	✷	✷	✷	✷	/	✷	/	/	5
Hassan et al.[40]	2006	✷	/	✷	✷	/	/	/	/	3
Changchien et al.[41]	2004	✷	✷	✷	✷	/	/	/	✷	5

Supplementary Table S1: Newcastle Ottawa Scale (NOS) risk of bias assessment for non-randomised studies

Certainty Assessment							No of Patients		Effect		Certainty	Importance
No of Studies	Study Design	Risk of Bias	Inconsistency	Indirectness	Imprecision	Other considerations	Neoadjuvant	Adjuvant	Relative (95% CI)	Absolute (95% CI)		
**Recurrence**												
4	Retrospective Cohort Study	Not serious	Serious[a]	Not serious	Serious[a]	None	80	131	**OR 0.22** (0.02 to 1.91)		⊕◯◯◯ Very Low	
**Overall 5-Year Survival**												
4	Retrospective Cohort Study	Not serious	Not serious	Not serious	Not serious	None	110	116	**OR 3.19** (1.37 to 7.40)		⊕⊕◯◯ Low	
**5 Year Disease Free Survival**												
4	Retrospective Cohort Study	Not serious	Serious[a]	Not serious	Serious[a]	None	95	116	**OR 1.25** (0.10 to 16.53)		⊕⊕◯◯ Low	

Certainty Assessment							No of Patients		Effect		Certainty	Importance
No of Studies	Study Design	Risk of Bias	Inconsistency	Indirectness	Imprecision	Other considerations	Local Excision	Radical Excision	Relative (95% CI)	Absolute (95% CI)		
**Length of Stay**												
5	Retrospective Cohort Study	Not serious	Not Serious	Not Serious	Not Serious	None	294	271	-	MD **6.74 lower** (6.92 lower to 6.56 lower)	⊕⊕◯◯ Low	
**Intraoperative Tumour Rupture**												
5	Retrospective Cohort Study	Not serious	Serious	Not serious	Serious	None	179	132	**OR 0.90** (0.35 to 2.34)		⊕◯◯◯ Very Low	
**Overall 5-Year Survival**												
4	Retrospective Cohort Study	Not serious	Serious	Not serious	Serious	None	243	241	**OR 1.31** (0.81 to 2.12)		⊕◯◯◯ Very Low	
**Recurrence**												
6	Retrospective Cohort Study	Not serious	Not serious	Not serious	Not Serious	None	156	133	**OR 0.67** (0.4 to 1.13)		⊕◯◯◯ Very Low	
**5 Year Disease Free Survival**												
4	Retrospective Cohort Study	Not serious	Not serious	Not serious	Not serious	None	126	114	**OR 1.10** (0.55 to 2.19)		⊕◯◯◯ Very Low	

CI: confidence interval; **MD:** mean difference; **OR**: odds ratio

Explanations:

a: High heterogeneity

Supplementary Table S2: GRADE Certainty of Evidence table
